# 

**Published:** 1910-03-26

**Authors:** 


THE HOSPITAL. March 26, 1910.

*Name* ..........................................

*Address* ..........................................

..........................................

This Coupon must accompany manuscript or contributions intended for The Hospital.

### THE MEDICAL LIBRARY.

Post Free.

"Medical History: From the Earliest Times." By E. T. Withington, M.A., M.B. Oxon ... 12s. 6d.

"The Consumptive Working Man. What Can the Sanatoria Do for Him?" By Noel D. Bardswell, M.D. ... ... ... ... ... ... 10s. 10d.

"Photomicrography." By Edmund Spitta, M.R.C.S. With 41 Half-tone Reproductions from original negatives and other Illustrations ... ... 12s. 0d.

Of all booksellers or of The Scientific Press, Limited, 28 and 29 Southampton Street, Strand, London, W.C.

### Gifts and Bequests.

Mr. Joseph Wade has bequeathed £100 to the Leeds General Infirmary.

The Royal Sea Bathing Hospital has received £126 from the Mercers' Company.

The Pewterers' Company has given 10 guineas to the Westminster Ophthalmic Hospital.

The London Fever Hospital has received £100 from Mrs. R. H. Raphael in memory of her husband.

Mr. S. Hope Morley has sent £105 to the Royal Hospital for Diseases of the Chest, City Road.

Galashiels Cottage Hospital has received intimation of £100 legacy from the late Rev. Robert Small.

The Ancoats Hospital has received £100 from Mr. Charles Behrens, Manchester, the Lord Mayor.

Mr. James Macdonald, of Birkenhead, Cheshire, has left £500 to the Ross Memorial Hospital, Dingwall, N.B.

Mr. Henry James Lubbock, of Lowndes Square, and of Lombard Street, has left £1,000 to St. George's Hospital.

Mr. Patrick Murphy, county Down, has left £5,000 each to the Mater Misericordiæ Hospital, Dublin, and the Royal Hospital, Belfast.

Mr. Allan Roskell, of Onslow Square, S.W., has bequeathed £500 each to St. George's Hospital, and the Hospital for Consumption, Brompton.

The Rev. William Stokes Shaw, M.A., Prebendary of Wells Cathedral, has left £10 to the Bath Eye Infirmary, and £10 to the Royal United Hospital, Bath.

Mr. Matthew Philip Christie, of Marylebone, has left £1,000 each to the Western General Infirmary, Stafford Street, Marylebone Road, N.W., and St. Mary's Hospital, Paddington.

Miss Elizabeth Catherine Moore, of South Hampstead, has left £50 each to King's College Hospital, the Middlesex Hospital, and her books to the Royal Hospital for Incurables, Putney, and the Hospital for Sick Children, Great Ormond Street, in equal shares.

Among the latest annual subscriptions received at the Bank of England for King Edward's Hospital Fund are: Messrs. Morgan, Grenfell, & Co., £500; Mr. Howard Morley, £105; Messrs. Robarts, Lubbock, & Co., £100; Messrs. Frank Bailey & Co., £52 10s.; Lord Farquhar, £50; staff at Fredk. Gorringe's (Limited), £31 4s. 11d.

Miss Jane Curtis Marshall, of Brighton, has left the ultimate residue of her estate, two-fortieths each to the Royal Hospital for Incurables, Putney; the Sussex Eye Hospital; the Brighton, Hove, and Sussex Throat and Eye Hospital; the Sussex County Homœopathic Dispensary, Brighton; the Royal Alexandra Hospital for Sick Children, Brighton; the Brighton, Hove, and Preston Dispensary; the Brighton, Hove, and Preston Provident Dispensary; and the Brighton and Hove Hospital for Women, Brighton; five-fortieths to the Sussex, East Hants, and Chichester General Infirmary and Dispensary; and six-fortieths to the Sussex County Hospital. The amount available for charitable purposes would appear to be about £20,000.

### THE QUESTION BOX.

**SPECIAL NOTICE.—Questions of interest affecting the honorary medical staff and hospital administration in all departments are invited. When any point arises we hope our readers will formulate it in a question and so co-operate to make this column of real value and interest. In this way the experience of the best-managed hospitals should be made helpful to the whole hospital world. We shall welcome the contribution of both questions and answers.**

**N.B.—The publication of an answer in this column does not necessarily preclude the publication of subsequent answers to the same question, for a question may involve points which require to be threshed out and fully discussed. Answers, if published, will be paid for at scale rates.**

### COST OF RATIONS FOR HOSPITAL STAFF.

Question: Is 1s. 2d. per day for rations only excessive in cost for a large Hospital staff?

### A Practical View.

The author of "Hospital Expenditure, the Commissariat," sends us the following:—

The rate of 1s. 2d. a head for catering for a large establishment is considerably in excess of what is necessary for maintaining a good standard of board. The housekeeper would be justified in spending this amount if provisions were bought at an exceptionally high rate, for in some institutions she has but little voice in arranging contracts or selecting supplies. Or, again, this amount might be justified if it were desired by the authorities that the food should be of a more luxurious or varied character than that usually provided in institutions. Such a rate of expenditure, for instance, would allow for poultry once a week, or for an unlimited supply of milk as a beverage, or for dessert of fresh or dried fruit daily. There would be no reason to object to such additions to the diet, provided only that the additional cost entailed were clearly defined, and that all were satisfied that good value was obtained for the money.

Under normal circumstances, with good buying, the following diet table for members of the staff works out at from 6s. to 6s. 6d. a week per head:—

Breakfast.—Porridge; bacon, eggs, or fish; white and brown bread; butter and marmalade. Tea or coffee. Apples or bananas.

Dinner.—Fresh roast or boiled meat or fish with egg sauce. Potatoes and other vegetables. Pudding, with choice of bread and cheese.

Afternoon tea.

Supper.—Cold meat or hot réchauffé, or macaroni, cheese, etc. Stewed fruit or bread and cheese or pudding or vegetables.

Every article of diet must, of course, be of good quality, frequently tested. There must be a well-paid and intelligent cook, and the meals must be served with precision and comfort. For dinner not less than 40 minutes should be allowed from the moment of sounding the second bell to the moment when the diners rise from the table. For other meals half an hour may suffice. It is necessary to emphasise these points in any consideration of the cost of catering, for the utmost care of the housekeeper will not suffice to feed the household well when the meals are ill-prepared, ill-served, and eaten in haste.

In cases where more than 6s. 6d. a week per head is spent over the food the housekeeper ought to be in a position to explain that the cost is due either to the rate at which the goods are purchased or to extras provided over and above the scale of living indicated above.

### THE PURCHASING OF PROVISIONS.

Question: Is it in the best interests of an institution for all provisions, etc., to be purchased and to be under the direct control of the matron's department or under the direct control of the Secretary's department?

### Appointments.

Mr. F. G. Killard Leavey, L.R.C.P., M.R.C.S., has been appointed house surgeon for one year to the Herefordshire General Hospital.

The following appointments for six months, from April 1 next, have been made at the Salford Royal Hospital, Manchester :—Resident surgical officer, Mr. A. C. Brown, M.B., Ch.B. St. Andrews; house physician, Mr. A. Gordon M'Leod, M.B., Ch.B. Edin.

The Council of Sheffield University has made the following appointments :—Mr. A. Garrick Wilson, F.R.C.S., to the post of Tutor in Surgery; Mr. Albert E. Bennee, M.B., Ch.B. Edin., to the post of Junior Demonstrator in the Department of Pathology.

### HOSPITALS AND THE RATES.

The following letter on hospitals and the rating system appeared in the *Times* recently: " . . . Owing to the inadequacy of our out-patient department to meet the increase of work carried out in it, we were compelled to provide new accommodation, and this, by the generosity of Mr. W. W. Astor, we have been enabled to do in the most efficient manner. In consequence of this improvement, our rates have this year been increased by £505 (from £795 16s. 5d. to £1,300 16s. 5d.). The new department brings us in no increase of revenue, but is, on the contrary, and necessarily, more expensive to maintain than was the old one. But for this rise in the rates our receipts for the year would have exceeded expenditure by £200; as it is there is a balance of £300 on the wrong side of our account. The whole purpose and tendency of our work is to save expense to the community. Can any justifiable reason be given why we should be mulcted by the community to the extent of £1,300 per annum for carrying out this purpose? We feel convinced that in the near future the whole question of the finances of the London hospitals will have to be inquired into by the Government, and our object in troubling you with this letter is to recommend that they should be exempted from municipal taxation, which would go far to relieve them from the great difficulties of their present financial position.

" Arthur Lucas, Chairman, Hospital for Sick Children, Great Ormond Street, W.C.

" John Murray, Vice-Chairman."

### BOOKS RECEIVED.

School Hygiene Publication Co., Ltd.
"School Hygiene." Nos. 1 and 2. Vol. I.

Messrs. H. K. Lewis.
" Some Common Remedies and their use in Practice." By Eustace Smith, M.D.

J. B. Lippincott Co.
" International Clinics." Vol. IV., 19th Series.

J. Nisbet and Co.
" Nisbet's Medical Directory," 1910.

Macmillan.
" The Highways and Byways of Middlesex." By H. Thomson.

W. B. Clive.
" Matriculation Directory." No. 54, January 1910.

E. and S. Livingstone (Edinburgh).
" Nurses' Guide to Prescription Reading." By J. G. H.

George Bell and Sons.
" Hoblyn's Dictionary of Terms used in Medicine." 14th Ed.

G. P. Putnam's Sons.
" A Quiz Book of Nursing." By Amy E. Pope and Thirza A. Pope.

John Murray.
" Health Progress and Administration in the West Indies." By Sir Rubert Boyce, F.R.S.
" Sinai and Palestine." By A. P. Stanley, D.D.
" River Amazons." By H. W. Bates.

Mackie and Co., Ltd.
" Dental Register," 1910.
" Medical Register," 1910.

Kegan Paul, Trench, Trubner and Co.

" Psychic Healing."

J. H. Glaisher.
" Mind and Health." By Edwin Ash, M.D.

**The Institution Library.**

" Hospital Expenditure: The Commissariat." For the use of Managers ... ... ... ... ... 2s. 6d.
" The District Nursing Association; Hints on how to Start it." (Net, post free.) ... ... ... 1s. 1d.
" A Legal Handbook for the use of Hospital Authorities." ... ... ... ... ... ... ... ... 2s. 6d.
" Burdett's Hospitals and Charities," 1909. (Net, post free) ... ... ... ... ... ... ... 7s. 11d.
Ledgers, Charts, Case-papers, and every kind of Institutional Literature.

Of all booksellers or of The Scientific Press, Limited, 28 and 29 Southampton Street, Strand, London, W.C.

